# Calcium-Dependent Ion Channels and the Regulation of Arteriolar Myogenic Tone

**DOI:** 10.3389/fphys.2021.770450

**Published:** 2021-11-08

**Authors:** William F. Jackson

**Affiliations:** Department of Pharmacology and Toxicology, College of Osteopathic Medicine, Michigan State University, East Lansing, MI, United States

**Keywords:** ion channels, calcium ions, arterioles, microcirculation, vascular smooth muscle, endothelial cells

## Abstract

Arterioles in the peripheral microcirculation regulate blood flow to and within tissues and organs, control capillary blood pressure and microvascular fluid exchange, govern peripheral vascular resistance, and contribute to the regulation of blood pressure. These important microvessels display pressure-dependent myogenic tone, the steady state level of contractile activity of vascular smooth muscle cells (VSMCs) that sets resting arteriolar internal diameter such that arterioles can both dilate and constrict to meet the blood flow and pressure needs of the tissues and organs that they perfuse. This perspective will focus on the Ca^2+^-dependent ion channels in the plasma and endoplasmic reticulum membranes of arteriolar VSMCs and endothelial cells (ECs) that regulate arteriolar tone. In VSMCs, Ca^2+^-dependent negative feedback regulation of myogenic tone is mediated by Ca^2+^-activated K^+^ (BK_Ca_) channels and also Ca^2+^-dependent inactivation of voltage-gated Ca^2+^ channels (VGCC). Transient receptor potential subfamily M, member 4 channels (TRPM4); Ca^2+^-activated Cl^−^ channels (CaCCs; TMEM16A/ANO1), Ca^2+^-dependent inhibition of voltage-gated K^+^ (K_V_) and ATP-sensitive K^+^ (K_ATP_) channels; and Ca^2+^-induced-Ca^2+^ release through inositol 1,4,5-trisphosphate receptors (IP_3_Rs) participate in Ca^2+^-dependent positive-feedback regulation of myogenic tone. Calcium release from VSMC ryanodine receptors (RyRs) provide negative-feedback through Ca^2+^-spark-mediated control of BK_Ca_ channel activity, or positive-feedback regulation in cooperation with IP_3_Rs or CaCCs. In some arterioles, VSMC RyRs are silent. In ECs, transient receptor potential vanilloid subfamily, member 4 (TRPV4) channels produce Ca^2+^ sparklets that activate IP_3_Rs and intermediate and small conductance Ca^2+^ activated K^+^ (IK_Ca_ and sK_Ca_) channels causing membrane hyperpolarization that is conducted to overlying VSMCs producing endothelium-dependent hyperpolarization and vasodilation. Endothelial IP_3_Rs produce Ca^2+^ pulsars, Ca^2+^ wavelets, Ca^2+^ waves and increased global Ca^2+^ levels activating EC sK_Ca_ and IK_Ca_ channels and causing Ca^2+^-dependent production of endothelial vasodilator autacoids such as NO, prostaglandin I_2_ and epoxides of arachidonic acid that mediate negative-feedback regulation of myogenic tone. Thus, Ca^2+^-dependent ion channels importantly contribute to many aspects of the regulation of myogenic tone in arterioles in the microcirculation.

## Introduction

Arterioles are prominent resistance vessels that regulate blood flow to and within tissues and organs; determine capillary blood pressure and fluid exchange in the microcirculation; and contribute to the regulation of systemic blood pressure (Renkin, 1984). A defining characteristic of arterioles is pressure-dependent myogenic tone, the steady state vascular smooth muscle cell (VSMC) contractile activity that is induced and maintained by pressure-dependent mechanisms (Jackson, 2020, 2021). Myogenic tone sets resting arteriolar internal diameter such that these microvessels can dilate or constrict to maintain homeostasis by meeting the blood flow and pressure needs of the tissues and organs that they perfuse.

**Figure 1:**
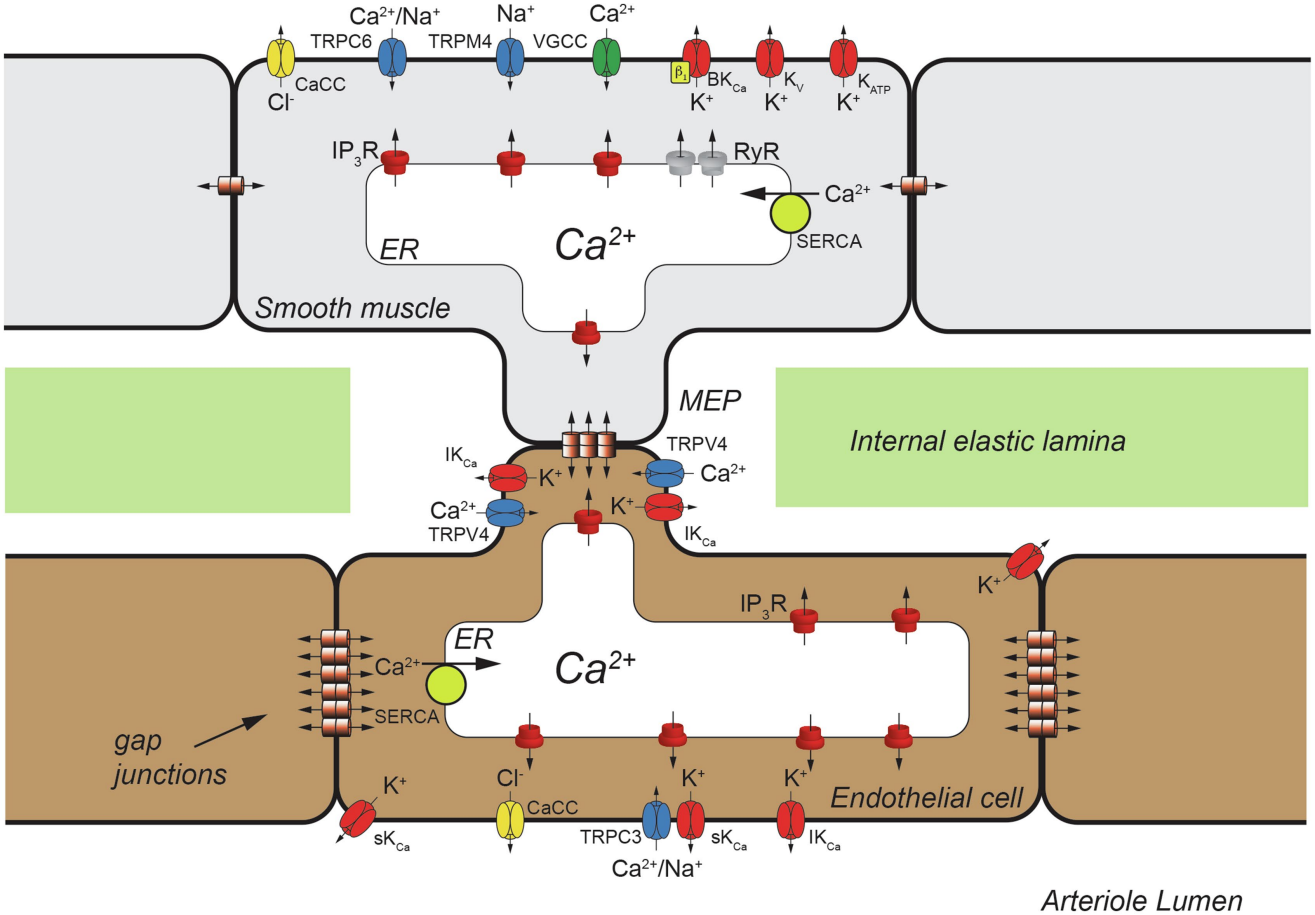
Schematic representation of a cross section of one wall of an arteriole showing a myoendothelial projection (MEP) passing through a hole in the internal elastic lamina (IEL). Heterocellular gap junctions are present allowing electrical and chemical (Ca^2+^, IP_3_, etc.) communication between ECs and VSMCs. Also shown are homocellular (EC-EC and VSMC-VSMC) gap junctions that also allow electrical and chemical communication as shown. Only a few classes of ion channels expressed by arteriolar VSMCs and ECs are shown for clarity. TRPC6, transient receptor potential channel C family member 6; CaCC, Ca^2+^-activated Cl^−^ channels; TRPM4, transient receptor potential channel melanostatin family member 4; VGCC, voltage-gated Ca^2+^ channels, BK_Ca_, large-conductance Ca^2+^-activated K^+^ channels; K_V_, voltage-gated K^+^ channels; K_ATP_, ATP-sensitive K^+^ channels; IP_3_R, inositol 1,4,5 trisphosphate receptor; RyR, ryanodine receptor; SERCA, smooth endoplasmic reticulum Ca^2+^ ATPase; IK_Ca_, intermediate-conductance Ca^2+^-activated K^+^ channel; TRPV4, Transient Receptor Potential Vanilloid-family 4 channels; TRPC3, transient receptor potential channel C family member 3; sK_Ca_, small-conductance Ca^2+^-activated K^+^ channel.

Arterioles express numerous ion channels that are essential to their function (Figure 1). Plasma membrane and endoplasmic reticulum (ER) ion channels in VSMCs are a major source of Ca^2+^ triggering contractile machinery activation and increased arteriolar tone (vasoconstriction). In endothelial cells (ECs), ion channels provide a key Ca^2+^source controlling EC autacoid production including prostacyclin (PGI_2_), nitric oxide (NO) and epoxides of arachidonic acid (EETs; Jackson, 2016). Intracellular Ca^2+^ also controls gene expression and cell proliferation in VSMCs (Cartin et al., 2000; Stevenson et al., 2001; Barlow et al., 2006) and in ECs (Quinlan et al., 1999; Nilius and Droogmans, 2001; Munaron, 2006; Minami, 2014). Ion channels play a major role in cell volume regulation in all cells (Hoffmann et al., 2009). Finally, ion channels help set and modulate VSMC and EC membrane potential (Jackson, 2016, 2020, 2021; Tykocki et al., 2017). Membrane potential, in turn, regulates the open state probability of voltage-gated Ca^2+^ channels (VGCCs) which provide a major source of activator Ca^2+^ in VSMCs (Tykocki et al., 2017), but probably not most ECs (Jackson, 2016). The electrochemical gradient for diffusion of Ca^2+^ and other ions depends on membrane potential in all cells (Tykocki et al., 2017). Membrane potential also has been proposed to affect Ca^2+^ release from ER Ca^2+^ stores and may influence the Ca^2+^ sensitivity of Ca^2+^-dependent processes [see (Tykocki et al., 2017) for references]. Lastly, membrane potential serves as an essential signal for cell–cell communication, because VSMCs and ECs express both homocellular and heterocellular gap junctions allowing electrical and chemical communication among cells in the arteriolar wall (de Wit and Griffith, 2010; Bagher and Segal, 2011; Dora and Garland, 2013; Garland and Dora, 2017; Schmidt and de Wit, 2020). Thus, arteriolar function critically depends on ion channels.

Calcium-dependent ion channels in both VSMCs and ECs play a central role in the generation and modulation of myogenic tone and maintenance of homeostasis (Figure 1). These channels provide both positive- and negative-feedback control of intracellular Ca^2+^ in VSMCs that allows fine tuning of arteriolar tone as will be outlined in Section VSMC Ca^2+^-Dependent Ion Channels, below.

The arteriolar endothelium provides negative-feedback signals to overlying VSMCs through Ca^2+^-dependent autacoid production and direct electrical communication *via* myoendothelial gap junctions (MEGJs; Lemmey et al., 2020). Endothelial Ca^2+^-dependent ion channels contribute to these processes (Figure 1) as outlined in Section Endothelial Ca^2+^-Dependent Ion Channels and Arteriolar Tone, below.

Section Integration of Ca^2+^-Dependent Ion Channels Into the Mechanisms Underlying Pressure-Induced Myogenic Tone then will integrate the VSMC and EC Ca^2+^-dependent ion channels into the mechanisms that establish, maintain, and modulate pressure-dependent myogenic tone in resistance arteries and arterioles.

## Vsmc Ca^2+^-Dependent Ion Channels

Arteriolar VSMCs express at least six different Ca^2+^-dependent ion channels (Tykocki et al., 2017) that participate in the generation, maintenance and modulation of myogenic tone. Large-conductance Ca^2+^-activated K^+^ (BK_Ca_) channels provide negative-feedback regulation of myogenic tone. Ryanodine receptors (RyRs) can be both inhibitory (negative feedback) or excitatory (positive feedback) dependent on where in the ER they are expressed and with which ion channels they interact. Inositol 1,4,5-trisphosphate receptors (IP_3_Rs), transient-receptor potential melanostatin member 4 (TRPM4) channels, Ca^2+^-activated Cl^−^ channels (CaCCs) and transient receptor potential polycystin-family member 1 [TRPP1 (PKD2)] channels are excitatory and contribute to the positive-feedback regulation of myogenic tone. In addition, VGCCs (Shah et al., 2006), voltage-gated K^+^ (K_V_) channels (Gelband et al., 1993; Ishikawa et al., 1993; Gelband and Hume, 1995; Post et al., 1995; Cox and Petrou, 1999) and ATP-sensitive K^+^ (K_ATP_) channels (Wilson et al., 2000) are inhibited in a Ca^2+^-dependent fashion and will be briefly discussed.

### VSMC BK_Ca_ Channels and the Regulation of Arteriolar Tone

**Figure 2:**
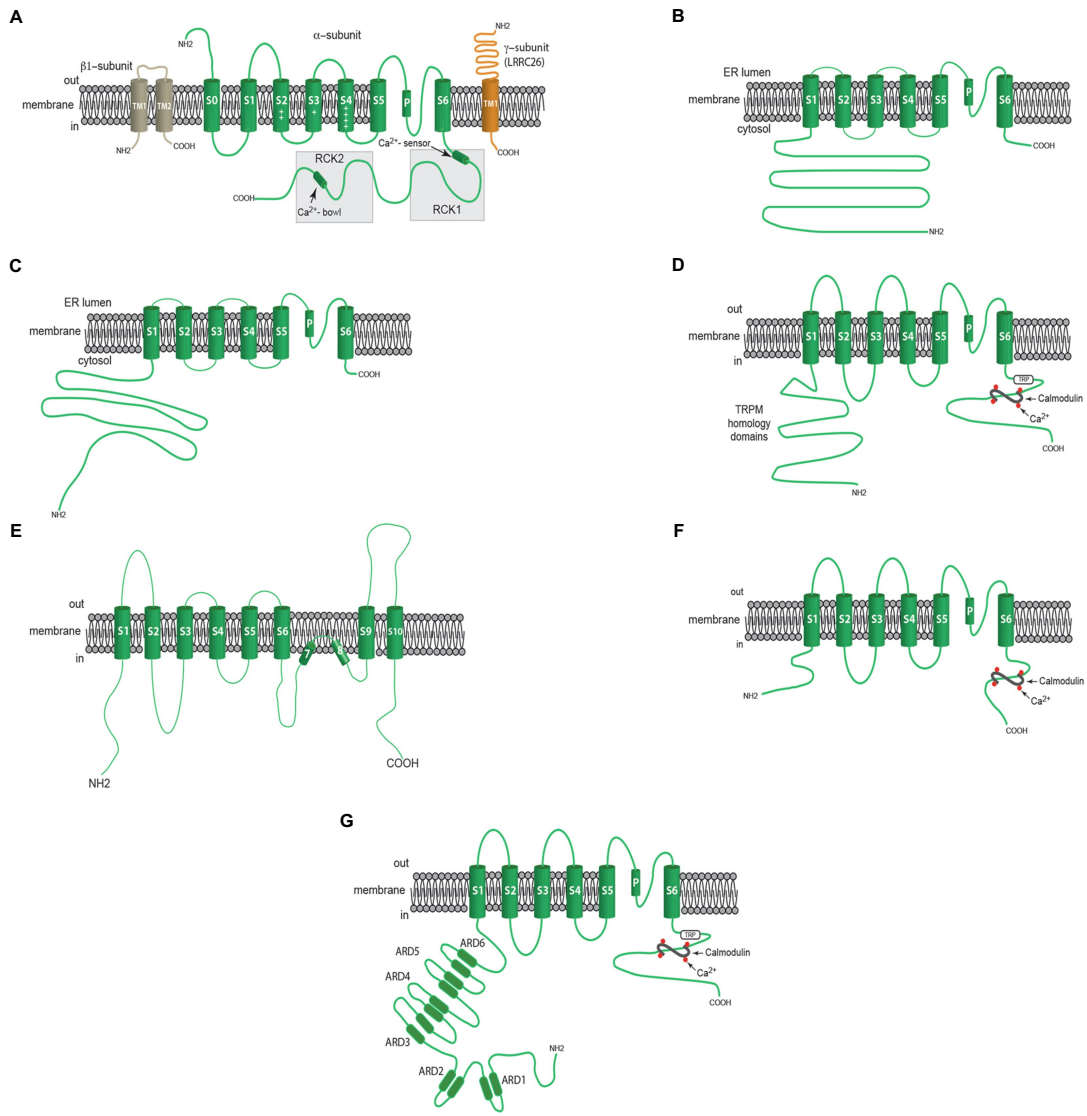
Membrane topology of Ca^2+^-dependent ion channels involved in the regulation of myogenic tone. **(A)** Components of VSMC BK_Ca_ channels including a β_1_- subunit with two membrane-spanning domains, one pore-forming α-subunit with seven membrane-spanning domains and a γ-subunit (LRRC26, for example) with one membrane-spanning domain. **(B)** Shows one α-subunit of an RYR with a large cytosolic N-terminal domain, 6 membrane spanning domains and a short C-terminal sequence. **(C)** Shows one α-subunit of an IP_3_R with a large cytosolic N-terminal domain, 6 membrane spanning domains and a short C-terminal sequence. **(D)** Shows one α-subunit of a TRPM4 channel including an N-Terminal domain with a TRPM homology sequence, 6 membrane spanning domains, and a C-terminal domain containing a TRP sequence and binding sites for calmodulin. **(E)** Shows one α-subunit of ANO1 (TMEM16A) CaCC with 10 membrane spanning domains. **(F)** Shows α-subunit of either sK_Ca_ or IK_Ca_ channels with 6 membrane spanning domains and a C-terminal domain with bindings sites for calmodulin. **(G)** Shows one α-subunit of a TRPV4 channel with N-terminal sequence containing ankyrin repeat domains (ARDs), 6 membrane spanning domains and a C-terminal domain with TRP sequence and calmodulin binding sites. See text for more information.

Arteriolar VSMCs express BK_Ca_ channels that provide negative-feedback regulation of myogenic tone (Figure 1). Both membrane depolarization and increases in intracellular Ca^2+^ activate BK_Ca_ (Tykocki et al., 2017), and because of their large conductance (~200 pS), they powerfully dampen the excitation of VSMCs, preventing vasospasm. BK_Ca_ channels consist of a tetramer of K_Ca_1.1 α-pore-forming subunits (gene=KCNMA1) which have seven transmembrane spanning domains (Meera et al., 1997; Figure 2A). Voltage is sensed by positively charged amino acids in membrane spanning domains S2, S3, and S4 (Ma et al., 2006; Figure 2A), while Ca^2+^ is sensed by two regulator of conductance of K^+^ (RCK) domains (RCK1 and RCK2) in the long, cytosolic C-terminus of the α-subunit (see (Hoshi et al., 2013a) for references; Figure 2A).

Vascular smooth muscle cells express both β and γ subunits that modulate the function of the BK_Ca_ channel α-pore-forming subunits (Figure 2A). The primary β subunits in VSMCs are β1 (KCNMB-1, K_Ca_β1; Tykocki et al., 2017; Figure 2A). These subunits modulate channel gating kinetics and increase the Ca^2+^ sensitivity of the α-subunit (McCobb et al., 1995; McManus et al., 1995; Meera et al., 1996; Tseng-Crank et al., 1996). They also are dynamically trafficked to the cell membrane from Rab11A-positive recycling endosomes, providing the ability of VSMCs to tune BK_Ca_ channel function (see (Leo et al., 2014, 2017) for details). The expression of β1-subunits may be downregulated during disease states like hypertension (Amberg et al., 2003; Tajada et al., 2013) and diabetes (McGahon et al., 2007), decreasing the ability to activate VSMC BK_Ca_ channels, increasing myogenic tone. The BK_Ca_ channel agonists dehydrosoyasaponin I (McManus et al., 1995) and 17β-estradiol require expression of β1-subunits (Valverde et al., 1999). Thus, β1-subunits control the Ca^2+^ sensitivity and the pharmacology of BK_Ca_ channels in VSMCs.

Arteriolar VSMC BK_Ca_ channels have a high Ca^2+^ setpoint requiring >3μM cytosolic Ca^2+^ ([Ca^2+^]_in_) to open at negative, physiological membrane potentials (−30 to −40mV; Jackson and Blair, 1998). For reference, global [Ca^2+^]_in_ measured with Fura-2 in arterioles with myogenic tone is on the order of 300–400nM (Brekke et al., 2006). Patch clamp studies have shown that arteriolar BK_Ca_ channels are silent when VSMCs are dialyzed with solutions containing 300nM [Ca^2+^]_in_ (Jackson, 1998), consistent with a high [Ca^2+^]_in_ threshold for their activation. The high Ca^2+^ setpoint (threshold) in arteriolar VSMCs may be due to lower expression of the β_1_-subunits (Yang et al., 2009, 2013) and possible differences in expression of spliced variants of the α-pore-forming subunit (Nourian et al., 2014) compared to VSMCs in larger arteries.

There also are γ-subunits associated with BK_Ca_ channels that are leucine-rich-repeat-containing proteins (LRRCs; Yan and Aldrich, 2010; Almassy and Begenisich, 2012; Evanson et al., 2014; Gonzalez-Perez et al., 2014; Figure 2A). LRRCs allow activation of BK_Ca_ channels at negative membrane potentials, even in the absence of Ca^2+^, by shifting their voltage vs. activity relationships to the left (increasing their voltage-sensitivity), facilitating their negative feedback function (Yan and Aldrich, 2010; Gonzalez-Perez et al., 2014). The BK_Ca_ channel sensitivity to activation by docosahexaenoic acid (DHA) also is increased by LRRCs (Hoshi et al., 2013b). The role played by LRRCs in arteriolar VSMCs has not been studied.

BK_Ca_ channels provide strong negative feedback regulation of both pressure-induced and agonist-induced tone in resistance arteries and arterioles [see (Tykocki et al., 2017) for numerous references]. However, there is regional heterogeneity in the source of Ca^2+^ that activates BK_Ca_ channels in resistance arteries versus arterioles. In most resistance arteries, BK_Ca_ channels are controlled by Ca^2+^ sparks which represent the simultaneous release of Ca^2+^ from the ER through small, clustered groups of RyRs (Nelson et al., 1995). Vascular smooth muscle cells that utilize this mechanism of BK_Ca_ channel activation display the so-called spontaneous-transient-outward currents (STOCs): bursts of activity of small groups of BK_Ca_ channels coinciding with the RyR-based Ca^2+^ sparks [(Nelson et al., 1995), see (Tykocki et al., 2017) for additional references]. In VSMCs where this mechanism is active, pharmacological block of RyRs produces the same effect as block of BK_Ca_ channels.

In contrast to many larger resistance arteries, Ca^2+^ influx through VGCCs directly activates BK_Ca_ channels in skeletal muscle arteriolar VSMCs; RyRs are silent, at least under the conditions studied (Westcott and Jackson, 2011; Westcott et al., 2012). In resistance arteries immediately upstream from skeletal muscle arterioles, both RyR-dependent and VGCC-dependent control of BK_Ca_ channels is apparent (Westcott and Jackson, 2011; Westcott et al., 2012). These data suggest that there may be a spectrum of control mechanisms that are involved in Ca^2+^-dependent control of BK_Ca_ channels in the resistance vasculature. In cerebral penetrating arterioles, both RyRs and BK_Ca_ channels are silent at rest, but both can be activated by low pH (Dabertrand et al., 2012). The molecular mechanisms underlying pH-sensitive recruitment of RyR-control of BK_Ca_ channels has not been established. The mechanisms responsible for the differences in Ca^2+^ sources that control BK_Ca_ channels are not known, but likely relate to the number and location of BK_Ca_ channels expressed relative to RyRs, VGCCs and other ion channels.

### VSMC Ryanodine Receptors and Arteriolar Tone

Ryanodine receptors are composed of four, >500kDa subunits that form ryanodine-sensitive Ca^2+^ channels in ER membranes (Figure 2B; Van Petegem, 2015; Yan et al., 2015; Zalk et al., 2015). Increases in [Ca^2+^]_in_ from resting levels [~300nM in VSMCs with tone (Brekke et al., 2006).] up to ~10μM activate release of Ca^2+^ through RyRs, although high levels of [Ca^+^]_in_ (>10μM) are inhibitory (Tykocki et al., 2017). Ryanodine receptors also serve as scaffolds for a plethora of signaling proteins [see (Tykocki et al., 2017) for numerous references]. There are three isoforms of RyRs, RyR1, RyR2 and RyR3 [genes=RYR1, RYR2 and RYR3, respectively (Lanner et al., 2010)]: RyR1 is predominantly expressed in skeletal muscle, RyR2 is expressed in cardiac muscle and RyR3 is expressed in the brain and other tissues (Ledbetter et al., 1994; Giannini et al., 1995; Reggiani and te Kronnie, 2006). Vascular smooth muscle expresses multiple isoforms of RYRs with considerable vessel-to-vessel heterogeneity (Vallot et al., 2000; Yang et al., 2005; Salomone et al., 2009; Vaithianathan et al., 2010; Westcott and Jackson, 2011; Westcott et al., 2012). In VSMCs of skeletal muscle arterioles, RyR2 is predominate, and RyR1 is absent (Westcott et al., 2012).

Ryanodine receptors are highly regulated proteins that are modulated by phosphorylation, cellular redox status and interactions with many binding partners in addition to [Ca^2+^]_in_ (see Tykocki et al., 2017). The overall function of RyRs depends on exactly where they are located in cells and with which ion channels and other proteins they interact.

The elemental Ca^2+^ signal generated by RyRs is the Ca^2+^ spark which represents the simultaneous release of Ca^2+^ from small clusters of RyRs as noted in Section VSMC BK_Ca_ Channels and the Regulation of Arteriolar Tone. Calcium influx through VGCCs has been shown to indirectly regulate Ca^2+^ spark frequency and amplitude by effects on global [Ca^2+^]_in_ and ER Ca^2+^ store loading (Essin et al., 2007). Subsequent studies have shown that the magnitude of Ca^2+^ influx through the persistent activity of membrane clusters of VGCCs, that can be recorded as VGCC Ca^2+^ sparklets (Navedo et al., 2005; Amberg et al., 2007), controls the amplitude of Ca^2+^ sparks (Tajada et al., 2013). These data suggest that local influx of Ca^2+^ is a major determinant of RyR activity in VSMCs.

In skeletal and cardiac muscle, RyRs act in a positive-feedback manner through Ca^2+^-induced-Ca^2+^-release (CICR) to cause explosive release of Ca^2+^ from the ER and subsequent muscle contraction. In both skeletal muscle and cardiac muscle, Ca^2+^ sparks form the basis of this positive feedback process. A similar positive feedback role for Ca^2+^ sparks has been proposed for some arteriolar VSMCs (Curtis et al., 2004, 2008; Fellner and Arendshorst, 2005, 2007; Balasubramanian et al., 2007; Tumelty et al., 2007; Kur et al., 2013). In addition to Ca^2+^ sparks, RyRs can cooperate with IP_3_Rs and contribute to Ca^2+^ waves and the positive regulation of myogenic tone in some resistance arteries (Jaggar, 2001; Mufti et al., 2010, 2015; Westcott and Jackson, 2011; Westcott et al., 2012). In other VSMCs, RyR-dependent Ca^2+^ sparks may also act in an excitatory fashion by activating plasma membrane CaCCs producing the so-called spontaneous transient inward currents (STICs) that cause membrane depolarization, VGCC activation and an increase in tone (ZhuGe et al., 1998; Cheng and Lederer, 2008).

As outlined in Section VSMC BK_Ca_ Channels and the Regulation of Arteriolar Tone, in many resistance arteries upstream from the microcirculation, RyRs function as part of a negative-feedback process limiting VSMC excitability. In these vessels, RyR-dependent Ca^2+^ sparks are functionally coupled to BK_Ca_ channels producing membrane hyperpolarization, VGCC deactivation and a decrease in tone (Nelson et al., 1995; Jaggar et al., 1998; Cheng and Lederer, 2008).

However, in skeletal muscle (Westcott and Jackson, 2011; Westcott et al., 2012), cerebral (Dabertrand et al., 2012), and ureteral (Borisova et al., 2009) arterioles downstream from resistance arteries, RyRs are not active and do regulate myogenic tone. Low pH has been shown to recruit RyR-dependent Ca^2+^ sparks in cerebral arterioles, thereby activating BK_Ca_ channels and mediating dilation (Dabertrand et al., 2012). Whether RyRs can be recruited by pH or other conditions in skeletal muscle or ureteral VSMCs has not been studied.

The mechanisms responsible for the heterogeneity in RyR function are not known but most likely result from the specific pattern and magnitude of RyR isoform expression, their cellular localization, and the expression and localization of other ion channels (for example, CaCC vs. BK_Ca_ channels) in the plasma membrane over RyRs. This area of research should be explored in more detail in the future.

### VSMC IP_3_Rs and Arteriolar Tone

Inositol 1,4,5 trisphosphate receptors are homotetramers that, like RyRs, form large (~310kDa) Ca^2+^ release channels in ER membranes (Foskett et al., 2007; Figure 2C). There is one binding site for IP_3_ on each IP_3_R monomer (Foskett et al., 2007; Seo et al., 2012, 2015; Taylor et al., 2014).

Three isoforms of IP_3_Rs (IP_3_R1, IP_3_R2, and IP_3_R3) arise from three genes (ITPR1, ITPR2 and ITPR3 respectively; Foskett et al., 2007). There is regional heterogeneity in VSMC IP_3_R expression and multiple isoforms are usually expressed in a given VSMC (see (Narayanan et al., 2012) for review). In VSMCs from skeletal muscle resistance arteries and downstream arterioles, we have found expression of IP_3_R1>IP_3_R2 >>IP_3_R3 (Westcott et al., 2012).

Like RyRs, IP_3_Rs can be triggered to open by increases in [Ca^2+^]_in_, with IP_3_ affecting the sensitivity of the channels to CICR [see (Tykocki et al., 2017) for review]. In the presence of IP_3_, IP_3_Rs display a bell shaped [Ca^2+^]_in_-response relationship with high [Ca^2+^]_in_ (>1μM) inhibiting Ca^2+^ release through these channels (Tu et al., 2005). IP_3_Rs serve as amplifiers of Ca^2+^ signals generated by other ion channels. They have a number of protein binding partners that modulate their function including FKBP12 (MacMillan et al., 2005), RACK1 (Patterson et al., 2004; Foskett et al., 2007), ankyrin (Hayashi and Su, 2001), Homer (Tu et al., 1998; Foskett et al., 2007), Bcl family members (Bcl-x_L_, Mcl and Bcl-2; Li et al., 2007; Eckenrode et al., 2010) and, importantly, a number of TRPC channels including TRPC1 (Boulay et al., 1999), TRPC3 (Boulay et al., 1999; Kiselyov et al., 1999), TRPC4 (Mery et al., 2001), TRPC6 (Boulay et al., 1999) and TRPC7 (Vazquez et al., 2006), either directly (Boulay et al., 1999) or as a component of larger protein complexes (Yuan et al., 2003).

Vascular smooth muscle IP_3_Rs are essential for the initiation and maintenance of myogenic tone in resistance arteries (Osol et al., 1993; Gonzales et al., 2010, 2014; Garcia and Earley, 2011) and some, but not all arterioles (Jackson and Boerman, 2017). Three mechanisms have been proposed to account for pressure-dependent activation of IP_3_Rs in resistance arteries including angiotensin receptor-mediated (Gonzales et al., 2014), or integrin-mediated (Mufti et al., 2015) activation of PLCγ_1_, angiotensin receptor-mediated activation of PLCβ (Mederos y Schnitzler et al., 2008; Schleifenbaum et al., 2014), or mechanisms involving membrane depolarization-induced activation of G_q_-coupled receptors (Ganitkevich and Isenberg, 1993; del Valle-Rodriguez et al., 2003; Urena et al., 2007; Mahaut-Smith et al., 2008; Liu et al., 2009; Fernandez-Tenorio et al., 2010; Yamamura et al., 2012).

In contrast, myogenic tone in hamster cheek pouch arterioles (Jackson and Boerman, 2017) and in murine 4^th^-order mesenteric arteries (Mauban et al., 2015) does not depend on IP_3_ and activation of IP_3_Rs. Phospholipase-mediated hydrolysis of phosphatidylcholine and subsequent production of diacylglycerol was proposed to participate in the generation and maintenance of myogenic tone in murine 4^th^-order mesenteric arteries (Mauban et al., 2015).

Myogenic tone in rat cerebral resistance arteries is accompanied by an increase in the frequency of Ca^2+^ waves (Jaggar, 2001; Mufti et al., 2010, 2015) that involve both IP_3_Rs (Mufti et al., 2015) and RyRs (Jaggar, 2001; Mufti et al., 2010, 2015). Similarly, Ca^2+^ waves in skeletal muscle resistance arteries depend on both RyRs and IP_3_Rs (Westcott and Jackson, 2011; Westcott et al., 2012). In contrast, Ca^2+^ waves in downstream skeletal muscle arterioles depend only on Ca^2+^ release from IP_3_Rs (Westcott and Jackson, 2011; Westcott et al., 2012) that may amplify Ca^2+^ influx through VGCCs (Jackson and Boerman, 2018). However, in rat (Miriel et al., 1999) and mouse (Zacharia et al., 2007) mesenteric resistance arteries, Ca^2+^ waves were inhibited as myogenic tone developed. Thus, there appears to be regional heterogeneity in the role played by IP_3_R in the development and maintenance of myogenic tone. The mechanisms responsible for the heterogeneity in function of IP_3_Rs among blood vessels has not been established but likely stems from differences in the IP_3_R isoforms that are expressed; their localization and interactions with other proteins; and their proximity to other ion channels.

### VSMC Ca^2+^-Activated Cl^−^ Channels and Arteriolar Tone

VSMCs also express CaCCs that may contribute to myogenic tone. The protein anoctamin-1 (gene=ANO1), also known as transmembrane member 16A (TMEM16A), appears to be the molecular basis of CaCCs in VSMCs (Ji et al., 2019). This protein exists as a homodimer with each monomer having 10 membrane spanning domains (S1-S10), with the pore being formed by S3-S7 helices which also contains a Ca^2+^ binding domain (Ji et al., 2019; Figure 2E). TMEM16A demonstrates a synergistic dependence on voltage and Ca^2+^ to control its activity, with depolarization and increases in [Ca^2+^]_in_ leading to opening of these channels (Ji et al., 2019). In vascular smooth muscle, [Cl^−^]_in_ is elevated due to intracellular Cl^−^ accumulation from the activities of the Cl^−^/HCO_3_^−^ exchanger and the Na^+^/K^+^/Cl^−^ co-transporter (Matchkov et al., 2013). The elevated [Cl^−^]_in_ sets the equilibrium potential for Cl^−^ [−40 to −25mV, (Matchkov et al., 2013)] to be positive to the resting membrane potential [−45 to −30mV, (Tykocki et al., 2017)] of VSMCs that develop myogenic tone. Therefore, opening of a Cl^−^ channel results in an outward Cl^−^ current (an inward current in electrophysiological terms), membrane depolarization, activation of VGCCs and an increase in tone (Matchkov et al., 2013).

Calcium-activated chloride channels contribute to agonist-induced tone in a variety of arteries (Bulley and Jaggar, 2014). In addition, STICs carried by Cl^−^ and coupled to RyR-mediated Ca^2+^ sparks or IP_3_-based Ca^2+^ waves have been reported (Bulley and Jaggar, 2014). Cerebral resistance artery VSMCs express TMEM16A that are functionally coupled to transient receptor potential C-family member 6 (TRPC6) channels. Calcium influx through TRPC6 activates TMEM16A contributing to the membrane depolarization, VGCC activation and pressure-induced myogenic tone in these vessels (Bulley et al., 2012; Wang et al., 2016). In hamster cheek pouch arterioles, CaCCs appear to contribute to myogenic tone when VGCCs are active (Jackson, 2020), suggesting that CaCCs may be functionally coupled to VGCCs in those VSMCs. The molecular identity of CaCCs in hamster cheek pouch arteriolar VSMCs has not been established. Additional research on expression and function of CaCCs in resistance arteries and arterioles appears warranted.

### VSMC TRPM4 Channels and Arteriolar Tone

**Figure 3:**
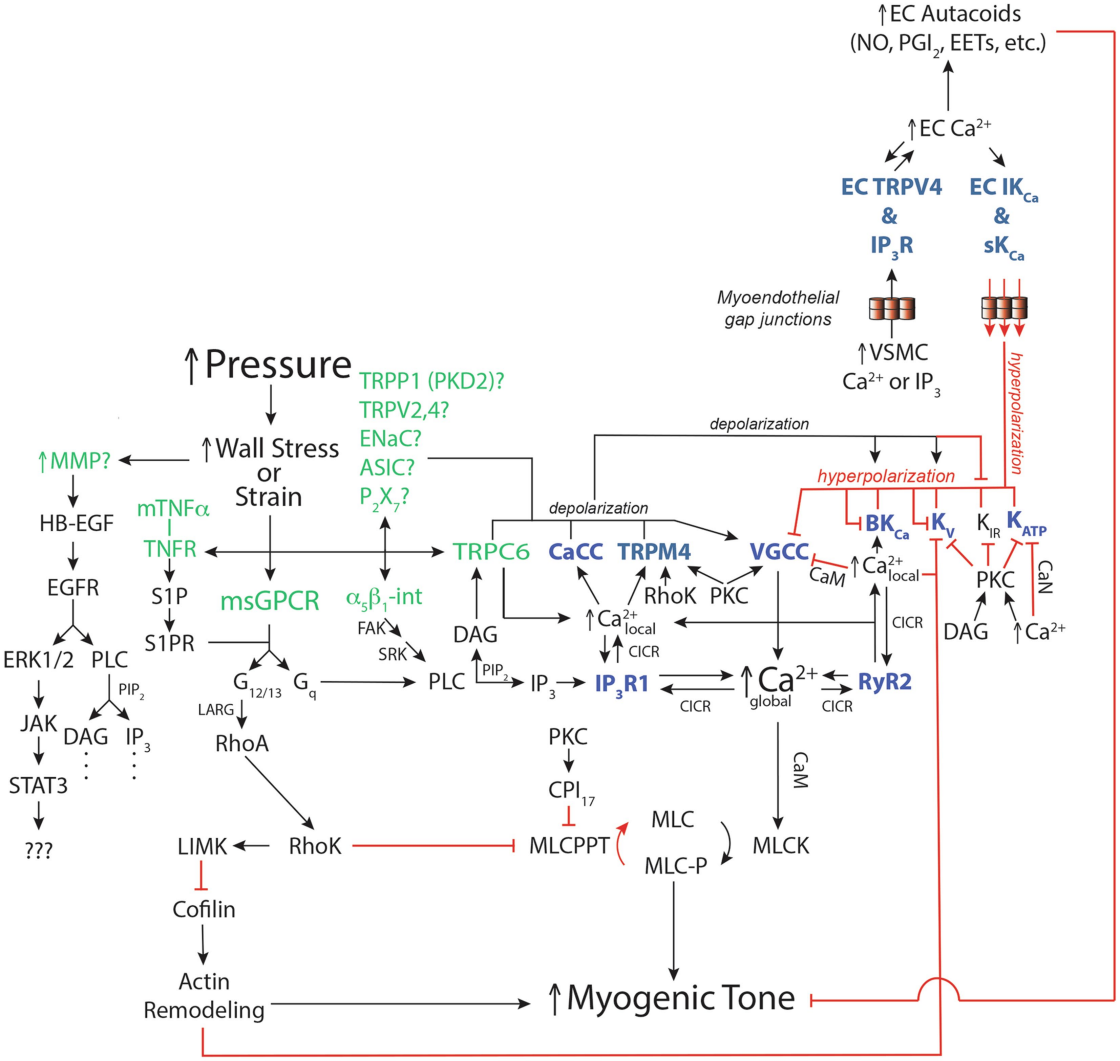
Ca^2+^-dependent ion channels and vascular smooth muscle signaling pathways for pressure-induced myogenic tone. Schematic diagram [modified from Jackson (2020), (2021)] of reported signaling pathways involved in myogenic tone in resistance arteries and arterioles highlighting the roles played by Ca^2+^-dependent ion channels. See Section Integration of Ca^2+^-Dependent Ion Channels Into the Mechanisms Underlying Pressure-Induced Myogenic Tone of text for more details and references. Green font color depicts putative mechanosensors in pressure-induced myogenic tone. Blue font color depicts Ca^2+^-dependent ion channels involved in regulation of myogenic tone. Black arrows show stimulation, increases or activation of signaling molecules, ion channels or enzymes that participate in myogenic tone. Red capped lines indicate inhibition, decreases or deactivation of signaling molecules, ion channels or enzymes involved in myogenic tone. EC, endothelial cell; VSMC, vascular smooth muscle cell; IK_Ca_, intermediate conductance Ca^2+^-activated K^+^ channel; sK_Ca_, small conductance Ca^2+^-activated K^+^ channel; MMP, matrix metalloproteinase; HB-EGF, heparin-bound epidermal growth factor; EGFR, Epidermal Growth Factor Receptor; ERK1/2, Extracellular-Signal-Related Kinases 1 or 2; JAK, Janus Kinase; STAT3, Signal Transducer and Activator of Transcription 3; mTNFα, membrane-bound Tumor Necrosis Factor α; TNFR, TNFα Receptor; S1P, Sphingosine-1-phosphate; S1PR, S1P Receptor; α_5_β_1_-int, α_5_β_1_ Integrin: FAK, Focal Adhesion Kinase; SRK, Src-related kinases; CaCC, Ca^2+^-activated Cl^−^ channel; TRPP1 (PKD2), Transient Receptor Potential Polycystin family member 1; TRPV2,4, Transient Receptor Potential Vanilloid-family 2 or 4 channels; ENaC, Epithelial Na^+^ Channel; ASIC, Acid Sensing Ion Channel; P_2_X_7_, P_2_X_7_ Purinergic Receptor; TRPC6, transient receptor potential C family member 6; TRPM4, transient receptor potential melanostantin member 4; VGCC, voltage-gated Ca^2+^ channel; BK_Ca_, large-conductance Ca^2+^-activated K^+^ channel; K_V_, voltage-gated K^+^ channel; K_IR_, inwardly-rectifying K^+^ channel; K_ATP_, ATP-sensitive K^+^ channel; msGPCR, mechanosensitive G-protein-coupled receptor; DAG, diacylglycerol; PKC, protein kinase C; PLC, phospholipase C; PIP_2_, phosphatidylinositol bisphosphate; IP_3_, inositol, 1,4,5 trisphosphate; IP_3_R1, IP_3_ receptor 1; RyR, ryanodine receptor; CICR, Ca^2+^-induced-Ca^2+^ release; LARG, Guanine Nucleotide Exchange Factor LARG; RhoA, small G-protein Rho; RhoK, Rho kinase; LIMK, LIM kinase; CPI_17_, C-kinase potentiated Protein phosphatase-1 Inhibitor; MLCPPT, myosin light-chain phosphatase; MLC, myosin light-chain; MLCK, myosin light-chain kinase; CaN, calcineurin; CaM, calmodulin.

VSMCs express many members of the transient receptor potential (TRP) family of ion channels that contribute to myogenic tone [see (Earley and Brayden, 2015; Tykocki et al., 2017) for more information; Figures 1, 3]. Of these, TRPM4 channels are Ca^2+^-activated and are essential for pressure-induced myogenic tone in cerebral resistance arteries (Gonzales et al., 2014). Like all TRP channels, the pore-forming subunit of TRPM4 channels has six transmembrane domains (S1–S6) which assemble as a tetramer to form a functional ion channel with residues in the intramembrane loop between S5 and S6 forming the channel’s pore (Earley and Brayden, 2015; Figure 2D). A conserved TRP domain located distal to S6 and a TRPM homology region in the NH2 terminus (Earley and Brayden, 2015) distinguish all members of the TRPM family (Earley and Brayden, 2015; Figure 2D). TRPM4 channels selectively conduct monovalent cations such that opening of these channels produces membrane depolarization due primarily to the influx of Na^+^ (Earley and Brayden, 2015). Calmodulin binding sites in the C-terminus of TRPM4 are essential for Ca^2+^-dependent activation and the Ca^2+^-sensitivity of these channels is increased by protein kinase C-dependent phosphorylation in their amino terminus (Earley, 2013). Rho kinase also has been reported to increase the Ca^2+^-sensitivity of TRPM4 channels in cerebral parenchymal arterioles (Li and Brayden, 2017).

In cerebral resistance arteries and arterioles, TRPM4 channels are part of the signal transduction pathway for pressure-dependent myogenic tone (Gonzales et al., 2014; Li et al., 2014; Li and Brayden, 2017; see Figure 3 and Section Integration of Ca^2+^-Dependent Ion Channels Into the Mechanisms Underlying Pressure-Induced Myogenic Tone for more details). In this scheme, TRPM4 channels are activated by release of Ca^2+^ through IP_3_Rs into the subplasmalemmal space (Gonzales et al., 2010), with the IP_3_Rs being activated by IP_3_, formed by mechanosensitive G-protein coupled receptor-mediated stimulation of phospholipase C (PLC)γ_1_, and Ca^2+^ entry through TRPC6 channels, likely activated by both pressure and PLCγ_1_-production of diacylglycerol (DAG; Gonzales et al., 2014; Figure 3). As noted above, in cerebral parenchymal arterioles, rho-kinase, which also is activated and contributes to myogenic tone, appears to modulate the Ca^2+^ sensitivity of TRPM4 channels (Li and Brayden, 2017; Figure 3). The Na^+^ entry through TRPM4 channels, along with the entry of Ca^2+^ and Na^+^ through TRPC6 channels produces membrane depolarization and activation of Ca^2+^ entry through VGCCs, hallmark elements of pressure-dependent myogenic tone (see (Tykocki et al., 2017) for numerous references; Figure 3). The role of TRPM4 in myogenic tone of vessels in other vascular beds has been questioned because global knockout of TRPM4 has no effect on pressure-induced tone in hind limbs of mice (Mathar et al., 2010). However, the details of the mechanisms responsible for pressure-induced tone in the TRPM4 knockout animals was not determined, such that compensation for the global knockout of TRPM4 channels may have occurred. Additional research on TRPM4 and myogenic tone appears warranted.

### VSMC TRPP1 (PKD2) Channels and Myogenic Tone

Another potentially Ca^2+^-activated ion channel that is involved in regulation of myogenic tone are TRPP1 (PKD2) channels. Similar to TRPM4 channels already described, TRPP1 channels are tetramers of 6 membrane spanning domains encoded by the PKD2 gene that have coiled-coil domains in their C-termini and a Ca^2+^-binding EF-hand motif that may be involved in Ca^2+^-dependent activation of these channels (Giamarchi and Delmas, 2007). The channels formed from TRPP1 are non-selective cation channels that conduct both Ca^2+^ and Na^+^ (Giamarchi and Delmas, 2007). The function of TRPP1 in regulation of myogenic tone is unclear. In murine mesenteric arteries, VSMC TRPP1 channels appear to inhibit myogenic tone (Sharif-Naeini et al., 2009), whereas in rat cerebral arteries VSMC TRPP1 channels significantly contribute to myogenic tone (Narayanan et al., 2013). Conditional knockout of TRPP1 from VSMCs decreases blood pressure and substantially reduces myogenic tone in murine skeletal muscle resistance arteries (Bulley et al., 2018). The plasma membrane expression of TRPP1 in VSMCs is controlled by recycling of sumoylated channels and SUMO1 modification of TRPP1 channels is required for pressure-induced myogenic tone (Hasan et al., 2019). How TRPP1 channels “fit” with other channels that have been shown to be involved in initiation and maintenance of myogenic tone (TRPC6 and TRPM4, for example) remains to be established. Nor has it been established that VSMC TRPP1 channels are activated by Ca^2+^ or that Ca^2+^-dependent activation is part of their role in pressure-dependent myogenic tone. It is known that TRPP1 channels can heterodimerize with other members of the TRP family (Giamarchi and Delmas, 2007) such that it is feasible that TRPP1 channels may be part of a multi-channel complex. Additional research will be required to determine how TRPP1 channels and all of the other VSMC ion channels implicated in the generation and maintenance of myogenic tone fit together.

### Inhibition of VSMC Ion Channels by Ca^2+^

Voltage-gated Ca^2+^ channels composed of CaV1.2 α-subunits (gene=CACNA1C) play a central role myogenic tone as these channels provide the main source of intracellular Ca^2+^, the primary trigger of VSMC contraction (Tykocki et al., 2017). Calcium-dependent inhibition of VGCCs is mediated by calmodulin that binds to the C-terminus of CaV1.2 channels that make up VSMC VGCCs (Shah et al., 2006). Thus, VGCCs themselves may contribute to the negative-feedback regulation of myogenic tone through this process (Figure 3).

Vascular smooth muscle cells express a diverse array of K_V_ channels that participate in the negative-feedback regulation of myogenic tone (Tykocki et al., 2017). Early studies showed Ca^2+^-dependent inhibition of K_V_ channel currents in VSMCs from large arteries (Gelband et al., 1993; Ishikawa et al., 1993; Gelband and Hume, 1995; Post et al., 1995; Cox and Petrou, 1999). However, the molecular identity of the K_V_ channel isoform that was inhibited was not identified: it was only suspected to be a channel inhibited by 4-amino pyridine (4-AP). Block of K_V_ channels by 4-AP appears to be Ca^2+^-dependent, making interpretation of 4-AP sensitivity difficult (Baeyens et al., 2014). It is well established that increased [Ca^2+^]_in_ inhibits K_V_7.2-7.5 channels *via* binding to calmodulin associated with these channels (Alaimo and Villarroel, 2018). K_V_7 channels contribute substantially to the regulation of myogenic tone in resistance arteries (Mackie et al., 2008; Greenwood and Ohya, 2009; Jepps et al., 2013; Cox and Fromme, 2016). Therefore, it is likely that at least some of the inhibitory effect of elevated [Ca^2+^]_in_ on whole-cell K_V_ currents is through inhibition of K_V_7 channels. Regardless, Ca^2+^-dependent inhibition of active K_V_ channels will cause membrane depolarization, activation of VGCCs and a further increase in [Ca^2+^]_in_ contributing to the positive-feedback regulation of myogenic tone (Figure 3). It should be noted that the density of K_V_ channels is such that Ca^2+^-dependent inhibition of these channels serves only to blunt the main, negative-feedback role that K_V_ channels play in the regulation of myogenic tone (Tykocki et al., 2017; Jackson, 2018).

Elevated [Ca^2+^]_in_ also inhibits ATP-sensitive K^+^ (K_ATP_) channels through Ca^2+^-dependent activation of the protein phosphatase, calcineurin (Wilson et al., 2000). These channels are active at rest in the microcirculation of a number of vascular beds (Tykocki et al., 2017). Closure of K_ATP_ channels by increased Ca^2+^ would contribute to membrane depolarization, activation of VGCCs, and a further increase in [Ca^2+^]_in_, a positive-feedback process that would increase myogenic tone (Figure 3).

## Endothelial Ca^2+^-Dependent Ion Channels And Arteriolar Tone

Numerous ion channels also contribute to EC function and to the modulation of myogenic tone (Jackson, 2016). Calcium-dependent ion channels in ECs include IP_3_Rs, small conductance Ca^2+^-activated K^+^ (sK_Ca_) channels, intermediate conductance Ca^2+^-activated K^+^ (IK_Ca_) channels, CaCCs, transient receptor potential vanilloid-family member 4 (TRPV_4_) channels and TRPP1 channels.

### EC IP_3_Rs and Arteriolar Tone

Endothelial cells express IP_3_Rs that contribute to the negative-feedback regulation of arteriolar myogenic tone. Early EC studies demonstrated that the initial increase in [Ca^2+^]_in_ in response to agonists of EC Gα_q_-coupled receptors resulted from Ca^2+^ release from ER stores (Hallam and Pearson, 1986; Colden-Stanfield et al., 1987; Busse et al., 1988; Schilling et al., 1992; Sharma and Davis, 1994, 1995). Subsequent studies pinpointed IP_3_Rs as the primary Ca^2+^ release channel involved in this response (Sharma and Davis, 1995; Cohen and Jackson, 2005).

Endothelial cells from arteries (Mountian et al., 1999, 2001; Grayson et al., 2004; Ledoux et al., 2008) and arterioles (Jackson, 2016) appear to express all three isoforms of IP_3_R. However, the dominant isoform may display regional- or species-dependent heterogeneity. For example, IP_3_R2 is the dominant IP_3_R expressed in mouse mesenteric artery ECs (Ledoux et al., 2008), whereas IP_3_R3 is the dominant IP_3_R in mouse cremaster muscle arteriolar ECs (Jackson, 2016). There is little information about the specific localization of IP_3_R in native arteriolar ECs. In both EC-VSMC co-cultures and in intact mouse cremaster arterioles, IP_3_R1 localizes at sites of MEGJs (Isakson, 2008). Similarly, in mouse mesenteric resistance arteries, EC IP_3_Rs cluster near holes in the internal elastic lamina (Ledoux et al., 2008), that are sites of myoendothelial projections (MEPs) and MEGJs (Sandow and Hill, 2000; Figure 1). Although the IP_3_R isoform(s) expressed in these IP_3_R clusters has not been identified, they were demonstrated to be the sites of EC Ca^2+^ pulsars, localized IP_3_-dependent Ca^2+^ events arising from clusters of IP_3_Rs in the ER that extend into MEPs (Kansui et al., 2008; Ledoux et al., 2008; Figure 1).

Myoendothelial projections and MEGJs are important signaling microdomains in resistance arteries and arterioles and contain a growing list of signaling proteins including IP_3_Rs (Kansui et al., 2008; Ledoux et al., 2008), IK_Ca_ channels (Sandow et al., 2006), TRPA1 channels (Earley et al., 2009a), TRPV4 channels (Sonkusare et al., 2012, 2014), anchoring proteins [e.g., AKAP150 (Sonkusare et al., 2014)], protein kinases [e.g., PKC (Sonkusare et al., 2014)], NO synthase (Straub et al., 2011; Wolpe et al., 2021), Na^+^/K^+^ ATPase (Dora et al., 2008) and other proteins (Straub et al., 2014; Wolpe et al., 2021; Figure 1). Calcium influx through TRPA1 and TRPV4, which produce small, localized Ca^2+^ events called Ca^2+^ sparklets, likely serves as the source of Ca^2+^ that actually triggers release of Ca^2+^ through IP_3_Rs to form both localized Ca^2+^ pulsars (Kansui et al., 2008; Ledoux et al., 2008), Ca^2+^ wavelets (Tran et al., 2012) and larger Ca^2+^ waves (Duza and Sarelius, 2004; Kansui et al., 2008) found in ECs of resistance arteries and arterioles. These Ca^2+^ events are then translated into several signals that are vasodilatory and tend to reduce or temper myogenic tone. Activation of EC sK_Ca_ and IK_Ca_ channels (Section EC sK_Ca_ and IK_Ca_ Channels and Arteriolar Tone, below) leads to EC hyperpolarization, which can be conducted through MEGJs to overlying VSMCs, deactivating VGCCs, reducing VSMC Ca^2+^ influx and decreasing myogenic tone (Figure 3). Endothelial cell IP_3_R Ca^2+^ signals also activate EC NO synthase and production of other EC autacoids (PGI_2_, EETs, H_2_O_2_, etc.) that diffuse to overlying VSMCS and reduce myogenic tone (Figure 3).

Global increases in [Ca^2+^]_in_ reported for ECs in intact resistance arteries or arterioles exposed to endothelium-dependent vasodilators (Dora et al., 1997; Marrelli, 2000; Cohen and Jackson, 2005; Socha et al., 2011) are a complicated blend of IP_3_R-mediated Ca^2+^ pulsars, Ca^2+^ wavelets and Ca^2+^ waves. Both the number and frequency of Ca^2+^ pulsars (Ledoux et al., 2008) and both synchronous (Duza and Sarelius, 2004; Socha et al., 2012) and asynchronous (Ledoux et al., 2008; Socha et al., 2012) Ca^2+^ waves are increased by endothelium-dependent vasodilators, such as acetylcholine (Ledoux et al., 2008; Socha et al., 2012) or adenosine (Duza and Sarelius, 2004). Additional research will be required to discover the precise IP_3_R isoform expression, location and function related to endothelium-dependent vasomotor activity and modulation of myogenic tone.

### Arteriolar ECs Do Not Express Functional RyRs

Early studies of ECs from large arteries provided evidence for expression of functional RyRs (Lesh et al., 1993; Graier et al., 1994, 1998; Ziegelstein et al., 1994; Rusko et al., 1995; Kohler et al., 2001b). In contrast, there is a lack of evidence for expression of RyRs in resistance artery and arteriolar ECs. Mouse mesenteric resistance artery ECs do not express mRNA for the three RyR isoforms, whereas transcripts for IP_3_Rs are readily detected (Ledoux et al., 2008). In addition, resting Ca^2+^ levels or acetylcholine-evoked Ca^2+^ events in mouse (Ledoux et al., 2008) or rat (Kansui et al., 2008) mesenteric resistance artery ECs are unaffected by concentrations of ryanodine that block RyRs. Similarly, mouse cremaster arteriolar ECs do not express message for RyRs (Jackson, 2016), and the RyR agonist, caffeine (10mM), has no effect on [Ca^2+^]_in_ in these ECs (Cohen and Jackson, 2005). These data do not support a role for RyRs in resistance artery or arteriolar EC Ca^2+^ signals.

### EC sK_Ca_ and IK_Ca_ Channels and Arteriolar Tone

Resistance artery and arteriolar ECs express both sK_Ca_ (K_Ca_2.3; gene=KCNN3) and IK_Ca_ (K_Ca_3.1; gene=KCNN4) channels (Kohler et al., 2001a; Eichler et al., 2003; Taylor et al., 2003; Sandow et al., 2006; Si et al., 2006; Grgic et al., 2009). These channels are a tetramer of six transmembrane domain subunits with cytosolic N- and C-termini (Adelman et al., 2012; Figure 2F). The ion conducting pore is formed from a pore loop between membrane spanning domains 5 and 6, as in voltage-gated K^+^ channels (Adelman et al., 2012). Calmodulin interacts with the intracellular C-terminus to gate opening of both channels (Xia et al., 1998; Fanger et al., 1999; Adelman et al., 2012; Sforna et al., 2018). The Ca^2+^ sensitivity of sK_Ca_ and IK_Ca_ channels is an order of magnitude higher than for BK_Ca_ channels. The threshold for activation by Ca^2+^ binding to calmodulin occurs at 100nM, 50% of maximal activation at 300nM and maximal activation at 1μM for both sK_Ca_ channels (Xia et al., 1998) and IK_Ca_ channels (Ishii et al., 1997). The distinct pharmacology of sK_Ca_ and IK_Ca_ channels has helped to define their function in intact vessels (Jackson, 2016).

Endothelial cell sK_Ca_ and IK_Ca_ channels are not distributed uniformly in the plasma membrane of ECs: IK_Ca_ channels cluster at MEPs (Sandow et al., 2006; Ledoux et al., 2008; Earley et al., 2009a), the site of MEGJs (Sandow and Hill, 2000), whereas sK_Ca_ channels are more distributed around the cell periphery (Sandow et al., 2006). Both channels appear to reside in macromolecular signaling complexes. At MEPs and near MEGJ’s, IK_Ca_ channels localize with IP_3_Rs (Ledoux et al., 2008), TRPA1 channels (Earley et al., 2009a), TRPV4 channels (Sonkusare et al., 2012, 2014), anchoring proteins [e.g., AKAP150 (Sonkusare et al., 2014)], protein kinases [e.g., PKC (Sonkusare et al., 2014)], nitric oxide synthase (Straub et al., 2011; Wolpe et al., 2021), Na^+^/K^+^ ATPase (Dora et al., 2008), likely G-protein coupled receptors (Sonkusare et al., 2014) and other proteins (Straub et al., 2014; Wolpe et al., 2021; Figure 1). Local Ca^2+^ signals through TRPA1 channels (Earley et al., 2009a), TRPV4 channels (Sonkusare et al., 2012, 2014), and/or IP_3_Rs (Ledoux et al., 2008) activate IK_Ca_ (and sK_Ca_) channels, leading to EC hyperpolarization and conduction of this signal to overlying VSMCs. Hyperpolarization then deactivates VSMC VGCCs reducing myogenic tone (Figure 3). EC hyperpolarization also may amplify Ca^2+^ influx through TRPA1 and TRPV4 channels by increasing the electrochemical gradient for Ca^2+^ influx (Qian et al., 2014).

Endothelial cell sK_Ca_ channels also exist in macromolecular signaling microdomains around the EC periphery. They are found in cholesterol-rich areas (caveolae or lipid rafts) and colocalize with caveolin-1 (Absi et al., 2007). Ca^2+^ influx through TRPC3 channels selectively activates sK_Ca_ channels in rat cerebral arteries (Kochukov et al., 2014), suggesting that TRPC3 and sK_Ca_ channels exist in the same microdomain. In mouse carotid arteries, sK_Ca_ channels are in caveolae adjacent to EC-EC gap junction plaques (Brahler et al., 2009). Conditional knockout of sK_Ca_ channels attenuates shear-stress-induced vasodilation in these arteries, suggesting that sK_Ca_ channel localization has functional consequences (Brahler et al., 2009). The respective EC localization of sK_Ca_ and IK_Ca_ channels and their signaling microdomains explain how these two channels mediate different facets of EC hyperpolarization and the regulation of myogenic tone (Crane et al., 2003; Si et al., 2006).

Because ECs are electrically coupled to VSMCs *via* MEGJs, resting membrane potential of ECs can impact myogenic tone. Resting EC membrane potential is determined, in part, by the activity of sK_Ca_ and IK_Ca_ channels. Overexpression of sK_Ca_ channels (which hyperpolarizes ECs) reduces myogenic tone of mesenteric resistance arteries (Taylor et al., 2003). In contrast, conditional knockout of sK_Ca_ channels has the opposite effect (EC depolarization and an increase in myogenic tone; Taylor et al., 2003). Consistent with these data, pharmacological inhibition of sK_Ca_ and IK_Ca_ channels, or both channels augment(s) myogenic tone in rat cerebral parenchymal arterioles (Cipolla et al., 2009; Hannah et al., 2011). Endothelial cell sK_Ca_ and IK_Ca_ channels seem to play a smaller role in modulating myogenic tone of larger cerebral resistance arteries, although they remain important in endothelium-dependent agonist-induced vasodilation (Cipolla et al., 2009). Nonetheless, sK_Ca_ and IK_Ca_ channels significantly contribute to EC-dependent negative-feedback regulation of myogenic tone.

Endothelium-dependent vasodilators that act through G_q_-coupled receptors also activate sK_Ca_ and IK_Ca_ channels. In some vessels, such as guinea-pig carotid artery (Corriu et al., 1996), rat mesenteric arteries preconstricted with phenylephrine (Crane et al., 2003) and porcine coronary arteries (Bychkov et al., 2002) both channels appear to be involved because block of both sK_Ca_ and IK_Ca_ channels is necessary to inhibit agonist-induced EC hyperpolarization. In contrast, IK_Ca_ channels mediate endothelium-dependent hyperpolarization and vasodilation in rat cerebral arteries (Marrelli et al., 2003) and in murine arteries and arterioles (Brahler et al., 2009). The reason for this heterogeneity in the roles played by sK_Ca_ and IK_Ca_ channels between vascular beds is not apparent and will require further research.

### EC BK_Ca_ Channels and Arteriolar Tone

The expression and function of BK_Ca_ channels in ECs remains debatable (Sandow and Grayson, 2009). As described for VSMCs, BK_Ca_ channels are activated by both voltage and Ca^2+^, have a much larger conductance (~250 pS) than sK_Ca_ and IK_Ca_ channels, do not require association with calmodulin, and display pharmacology distinct from sK_Ca_ and IK_Ca_ channels (Hoshi et al., 2013a; Tykocki et al., 2017). Cultured large artery ECs have been reported to express BK_Ca_ channels (see (Sandow and Grayson, 2009) for references). Native ECs isolated from hypoxic rats (Hughes et al., 2010; Riddle et al., 2011) or cholesterol depleted ECs (Riddle et al., 2011) express functional BK_Ca_ channels. In cultured ECs, BK_Ca_ channels are located in caveolae and caveolin inhibits their function (Wang et al., 2005). These studies open the possibility that EC BK_Ca_ channels are normally inhibited. Conversely, chronic hypoxia, and potentially other stresses or pathologies, that alter membrane lipid domains may upregulate EC BK_Ca_ channel function (Sandow and Grayson, 2009).

Electrophysiological studies of freshly isolated bovine coronary artery (Gauthier et al., 2002), mouse carotid artery (Brahler et al., 2009), and rat cerebral parenchymal arteriolar (Hannah et al., 2011) ECs found only sK_Ca_ channel and IK_Ca_ channel currents; no BK_Ca_ channel currents were detected. While it has been reported that ECs in freshly isolated rat cremaster arterioles express protein for BK_Ca_ channels (Ungvari et al., 2002), neither mRNA nor protein for this channel were detected in bovine coronary artery ECs (Gauthier et al., 2002). Murine skeletal muscle resistance artery and arteriolar ECs lack BK_Ca_ channel mRNA (Jackson, 2016). Thus, there may be regional or species heterogeneity in EC expression of BK_Ca_ channels. Additional research appears to be warranted to define if and where EC BK_Ca_ are expressed, how they are regulated and their function in the regulation of myogenic tone.

### EC Ca^2+^-Activated Cl^−^ Channels and Arteriolar Tone

Electrophysiological studies of bovine pulmonary artery and human umbilical vein ECs demonstrate the functional expression of CaCCs (Nilius et al., 1997; Zhong et al., 2000). Unlike VSMCs (see Section VSMC Ca^2+^-Activated Cl^−^ Channels and Arteriolar Tone), initial studies did not report expression of TMEM16A in ECs in lung sections (Huang et al., 2009; Ferrera et al., 2011). However, more recent studies have identified TMEM16A expression and function in human pulmonary artery ECs and have shown that over expression of these channels leads to EC dysfunction (Skofic Maurer et al., 2020). In hypertension, EC TMEM16A also contributes to endothelial dysfunction (Ma et al., 2017). TMEM16A is expressed in murine cerebral capillary ECs where it regulates membrane potential, Ca^2+^ signaling, proliferation, migration, and blood brain barrier permeability (Suzuki et al., 2020). Block of TMEM16A preserves blood brain barrier function after ischemic stroke (Liu et al., 2019). Hypoxia stimulates proliferation of brain capillary ECs *via* increased expression of TMEM16A (Suzuki et al., 2021). Hypoxia also increases expression of TMEM16A in mouse cardiac ECs (Wu et al., 2014).

The function of TMEM16A in arteriolar ECs related to regulation of myogenic tone is not clear. In murine capillary ECs, block of TMEM16A results in membrane hyperpolarization suggesting that in ECs, like in VSMCs (see Section VSMC Ca^2+^-Activated Cl^−^ Channels and Arteriolar Tone), activation of these CaCCs leads to membrane depolarization, counter to the effects of activation of EC sK_Ca_ and IK_Ca_ channels which produce robust EC hyperpolarization. Thus, it may be that CaCCs in ECs are part of a negative feedback mechanism to dampen membrane hyperpolarization induced by EC sK_Ca_ and IK_Ca_ channels when intracellular Ca^2+^ is elevated.

### EC TRPV4 and Regulation of Arteriolar Tone

Transient receptor potential vanilloid-family member 4 channels are another prominent Ca^2+^-modulated ion channel expressed in ECs (Sonkusare et al., 2012, 2014; Hong et al., 2018; Chen and Sonkusare, 2020). These channels are formed from a tetramer of six membrane spanning domain subunits, with the pore of the channel formed by a pore-loop between domains 5 and 6 like many other ion channels (Figure 2G). They conduct primarily Ca^2+^ and are activated by a diverse array of chemicals including EETs (Nilius et al., 2004). In ECs, TRPV4 channels exist in signaling complexes near MEGJ’s along with IK_Ca_ channels, IP_3_Rs and other proteins (Sonkusare et al., 2012, 2014; Hong et al., 2018; Chen and Sonkusare, 2020; Figures 1, 3). Intracellular Ca^2+^ potentiates the activation of TRPV4 channels through calmodulin that binds to the C-terminal region of this channel (Strotmann et al., 2003).

Endothelial TRPV4 channels mediate agonist-induced, endothelium-dependent vasodilation, particularly in arterioles where activation of these receptors leads to activation of IK_Ca_ channels, EC hyperpolarization and conduction of this hyperpolarization to overlying VSMCs to induce vasodilation (Marrelli et al., 2007; Earley et al., 2009b; Sonkusare et al., 2012, 2014; Zhang et al., 2013; Zheng et al., 2013; Du et al., 2016; Diaz-Otero et al., 2018; Figure 3). In addition, TRPV4 channels play a central role in myoendothelial negative-feedback that tempers vascular tone in the absence of an endothelial agonist. Agonist-induced activation of VSMC Gq-coupled receptors leads to a global increase in EC intracellular Ca^2+^(Dora et al., 1997; Schuster et al., 2001; Tuttle and Falcone, 2001; Jackson et al., 2008; Kansui et al., 2008) that contributes to the negative-feedback regulation of vascular tone (Lemmey et al., 2020). Studies in murine mesenteric resistance arteries have shown that endothelial TRPV4 channels are activated during this process through a mechanism involving Ca^2+^ release through IP_3_Rs, resulting in activation of IK_Ca_ channels blunting agonist-induced vasoconstriction (Hong et al., 2018; Figure 3). Similarly, studies in rat cremaster arterioles have shown that endothelial TRPV4 channels are activated at low intravascular pressure, leading to TRPV4 Ca^2+^ sparklets (localized [Ca^2+^]_in_ signals through small groups of TRPV4 channels), activation of IK_Ca_ channels and dampening of myogenic tone (Bagher et al., 2012). The precise signal that is communicated from VSMCs to ECs to initiate myoendothelial feedback remains in question, with data supporting Ca^2+^ as the signal (Garland et al., 2017) and other findings supporting IP_3_ as the signal (Tran et al., 2012; Hong et al., 2018). Additional research will be required to determine whether Ca^2+^ or IP_3_ mediates myoendothelial negative-feedback and whether there is heterogeneity among vessels in which signal (Ca^2+^ or IP_3_) is used.

### EC TRPP1 Channels and Myogenic Tone

Endothelial cells also express TRPP1 channels where they function in shear-stress dependent vasodilation (MacKay et al., 2020). Shear-stress-induced increases in EC [Ca^2+^]_in_ that activate sK_Ca_ channels, IK_Ca_ channels and EC nitric oxide synthase were shown to be substantially impaired by conditional knockout of EC TRPP1 with no change in Ca^2+^ signals activated by muscarinic receptor activation (MacKay et al., 2020). Calcium-dependent activation of TRPP1 channels was not established in these studies, so [Ca^2+^]_in_ modulation of these channels in ECs and their role in regulating myogenic tone other than when activated by shear-stress remains to be established.

## Integration Of Ca^2+^-Dependent Ion Channels Into The Mechanisms Underlying Pressure-Induced Myogenic Tone

As outlined in Sections above, Ca^2+^-dependent ion channels in VSMCs and ECs are involved in the initiation, maintenance and modulation of pressure-induced myogenic tone. Figure 3 integrates this information into a working model with the function of VSMC and EC Ca^2+^-dependent ion channels highlighted.

### Pressure-Dependent Activation of Mechanosensors Leads to Formation of IP_3_ and DAG

Multiple mechano-sensors of wall stress (or strain) initiate the myogenic response culminating in steady-state myogenic tone (Figure 3). Putative sensors (in green font in Figure 3) include: several G-protein coupled receptors (Brayden et al., 2013; Narayanan et al., 2013; Schleifenbaum et al., 2014; Storch et al., 2015; Kauffenstein et al., 2016; Mederos et al., 2016; Hong et al., 2017; Pires et al., 2017; Chennupati et al., 2019), various cation channels (Welsh et al., 2002; Jernigan and Drummond, 2005; Gannon et al., 2008; VanLandingham et al., 2009; Narayanan et al., 2013; Nemeth et al., 2020), integrins (Davis et al., 2001; Martinez-Lemus et al., 2005; Colinas et al., 2015), matrix metalloproteinases and epidermal growth factor receptors (EGFR; Lucchesi et al., 2004; Amin et al., 2011); and membrane-bound tumor necrosis factor α (mTNF α), TNF α receptor (TNFR) and downstream sphingosine-1-phosphate (S1P) signaling (Kroetsch et al., 2017; Figure 3). Pressure-dependent stimulation of these putative mechano-sensors activates phospholipase C (PLC) catalyzing hydrolysis of membrane phosphatidyl inositol 4,5 bisphosphate (PIP_2_) to form IP_3_ and DAG (Figure 3).

### Activation of Plasma Membrane Ion Channels Produces Membrane Depolarization

Pressure- and likely DAG-induced activation of plasma membrane TRPC6 channels results in Ca^2+^ influx through these channels (Slish et al., 2002; Welsh et al., 2002). The resultant local [Ca^2+^]_in_ increase, along with IP_3_, activates IP_3_Rs to release Ca^2+^ from the ER, amplifying the local [Ca^2+^]_in_ increase. This subplasmalemmal increase in [Ca^2+^]_in_ then activates overlying plasma membrane TRPM4 channels. Calcium influx through TRPC6 channels also activates plasma membrane Ca^2+^-activated Cl^−^ channels (CaCCs; Bulley et al., 2012; Wang et al., 2016). The cation influx through TRPC6 and TRPM4 channels, and Cl^−^ efflux through CaCCs causes membrane depolarization (Figure 3). As noted in Section Pressure-Dependent Activation of Mechanosensors Leads to Formation of IP_3_ and DAG and shown in Figure 3, additional cation channels including TRPP1 channels may contribute to the pressure-induced depolarization.

### Membrane Depolarization Activates VGCC, Induces Ca^2+^ Influx and Stimulates VSMC Contraction

Membrane depolarization induced by ionic currents through TRPC6 channels, TRPM4 channels, CaCCs and other ion channels activates plasma membrane VGCCs resulting in Ca^2+^ influx. VGCC-mediated Ca^2+^ influx across the plasma membrane, along with IP_3_R-mediated Ca^2+^ release from ER Ca^2+^ stores, increases cytoplasmic (global) [Ca^2+^]_in_ levels, leading to calmodulin-mediated myosin light-chain kinase (MLCK) activation, phosphorylation of the myosin light-chains (MLC), actin-myosin cross-bridge formation, cross bridge cycling and an increase in myogenic tone (vasoconstriction; Cole and Welsh, 2011; Figure 3).

### K^+^ Channels Provide Negative Feedback to Dampen Myogenic Tone

Membrane depolarization-induced activation of VGCCs is inherently a positive-feedback process because the Ca^2+^ influx through these channels will itself lead to depolarization and further activation of VGCCs. This process is limited in VSMCs by activation of at least three negative-feedback processes. Membrane depolarization activates K_V_ channels, and membrane depolarization along with increased [Ca^2+^]_in_ activates BK_Ca_ channels. The K^+^ efflux through these two K^+^ channels (which by themselves would cause membrane hyperpolarization) blunts and limits depolarization-induced activation of VGCC (Figure 3; Jackson, 2017, 2020). Additional negative feedback arises from Ca^2+^-dependent inactivation of VGCCs (Shah et al., 2006; Figure 3).

### Parallel Activation of Protein Kinase C and Rho-Kinase

In addition to activating TRPC6 channels, the DAG formed from the activity of PLC along with elevated [Ca^2+^]_in_ activates protein kinase C (PKC) supporting the increase in tone by increasing the activity of TRPM4 channels (supporting depolarization) and VGCCs (promoting Ca^2+^ influx) while blunting the activity of several K^+^ channels (also supporting membrane depolarization; Jackson, 2020, 2021; Figure 3). The negative feedback involving K_V_ channels is blunted by Ca^2+^-dependent inhibition of these channels (Gelband et al., 1993; Ishikawa et al., 1993; Gelband and Hume, 1995; Post et al., 1995; Cox and Petrou, 1999; Figure 3). Ca^2+^-dependent activation of the protein phosphatase, calcineurin, inhibits ATP-sensitive K^+^ (K_ATP_) channels, limiting their activity and promoting depolarization (Wilson et al., 2000; Figure 3).

Stimulation of the mechano-sensors in vascular smooth muscle also activates the small G-protein rhoA, which, in turn, activates rho-kinase (Chennupati et al., 2019; Figure 3). Rho kinase phosphorylates a number of substrates that also support myogenic tone including inhibition of myosin light chain phosphatase (MLCPPT; Cole and Welsh, 2011), stimulation of actin cytoskeleton remodeling that accompanies activation of the contractile machinery (Loirand et al., 2006; Moreno-Dominguez et al., 2013), inhibition of K_V_ channels as a consequence of actin remodeling (Luykenaar et al., 2009) and increasing the Ca^2+^ sensitivity of TRPM4 channels (Li and Brayden, 2017; Figure 3). Activated PKC also may inhibit MLCPPT through phosphorylation of the inhibitory protein, CPI_17_ (Cole and Welsh, 2011; Figure 3).

### Endothelial Cells Contribute to the Negative-Feedback Regulation of Myogenic Tone

Endothelial cells lining resistance arteries and arterioles play a negative-feedback role, dampening myogenic tone both through the Ca^2+^-dependent production of vasodilator autacoids (PGI_2_, NO, EETS, etc.) and by conduction of Ca^2+^-dependent membrane hyperpolarization from the endothelium to overlying VSMCs *via* MEGJs (Figures 1, 3). Endothelial cells chemically and electrically converse with VSMCs through MEGJs that may form at myoendothelial projections that penetrate holes in the internal elastic lamina and contact the overlying VSMCs. Heterocellular gap junctions (MEGJs) between ECs and VSMCs form and allow small molecules (like IP_3_) and ionic currents (including Ca^2+^) to move between the cells. Pressure-induced increases in VSMC [Ca^2+^]_in_ or IP_3_ can pass to endothelial cells leading to EC IP_3_R-induced Ca^2+^ signals (Ca^2+^ pulsars and wavelets) that can increase the production of Ca^2+^-dependent EC vasodilator autacoids that feedback to the VSMCs reducing myogenic tone (Figure 3). In addition, increased EC [Ca^2+^]_in_ will activate EC sK_Ca_ and IK_Ca_ channels causing EC membrane hyperpolarization. Myoendothelial gap junctions allow this hyperpolarization to be passed from ECs to VSMCs, producing VSMC hyperpolarization, deactivation of VSMC VGCCs and reduced myogenic tone (Figure 3). Thus, the production of EC autacoids and EC membrane potential are both strongly dependent on the activity of Ca^2+^-dependent ion channels in the endothelium including IP_3_Rs, TRPV4 channels, sK_Ca_ channels and IK_Ca_ channels (Lemmey et al., 2020).

## Final Perspective

As outlined in this perspective, Ca^2+^-activated ion channels in both VSMCs and ECs contribute to the regulation of myogenic tone. However, there appears to be considerable heterogeneity in the specific details of their roles in this process among vessels in different vascular beds around the body. The mechanisms responsible for this heterogeneity remains to be established. It is also clear that there is a paucity of information about the cellular and molecular details surrounding which channels are expressed, their localization and their regulation relative to myogenic tone in arterioles around the body. Mesenteric and cerebral resistance artery ion channel expression and function has been well studied. However, we know relatively little about ion channel expression and function in the downstream arterioles in microcirculation, which is really the business end of the cardiovascular system. Future studies directed specifically at understanding control of ion channel expression and function in the microcirculation and how they vary among vascular beds in different organs is warranted.

## Author Contributions

WJ conceived, wrote, and edited this manuscript.

## Funding

Supported by National Heart, Lung and Blood Institute grants HL-137694 and PO1-HL-070687.

## Author Disclaimer

The content is solely the responsibility of the author and does not necessarily represent the official views of the National Institutes of Health.

## Conflict of Interest

The author declares that the research was conducted in the absence of any commercial or financial relationships that could be construed as a potential conflict of interest.

## Publisher’s Note

All claims expressed in this article are solely those of the authors and do not necessarily represent those of their affiliated organizations, or those of the publisher, the editors and the reviewers. Any product that may be evaluated in this article, or claim that may be made by its manufacturer, is not guaranteed or endorsed by the publisher.
